# The Potential of Sequential Fermentations in Converting C1 Substrates to Higher-Value Products

**DOI:** 10.3389/fmicb.2022.907577

**Published:** 2022-06-03

**Authors:** Christina Stark, Sini Münßinger, Frank Rosenau, Bernhard J. Eikmanns, Andreas Schwentner

**Affiliations:** ^1^Institute of Microbiology and Biotechnology, University of Ulm, Ulm, Germany; ^2^Institute of Pharmaceutical Biotechnology, University of Ulm, Ulm, Germany

**Keywords:** bio-based acetate, bio-based ethanol, synthesis gas, methanol, formate, sequential C1 fermentations, acetogens, biological gas-to-liquids

## Abstract

Today production of (bulk) chemicals and fuels almost exclusively relies on petroleum-based sources, which are connected to greenhouse gas release, fueling climate change. This increases the urgence to develop alternative bio-based technologies and processes. Gaseous and liquid C1 compounds are available at low cost and often occur as waste streams. Acetogenic bacteria can directly use C1 compounds like CO, CO_2_, formate or methanol anaerobically, converting them into acetate and ethanol for higher-value biotechnological products. However, these microorganisms possess strict energetic limitations, which in turn pose limitations to their potential for biotechnological applications. Moreover, efficient genetic tools for strain improvement are often missing. However, focusing on the metabolic abilities acetogens provide, they can prodigiously ease these technological disadvantages. Producing acetate and ethanol from C1 compounds can fuel *via* bio-based intermediates conversion into more energy-demanding, higher-value products, by deploying aerobic organisms that are able to grow with acetate/ethanol as carbon and energy source. Promising new approaches have become available combining these two fermentation steps in sequential approaches, either as separate fermentations or as integrated two-stage fermentation processes. This review aims at introducing, comparing, and evaluating the published approaches of sequential C1 fermentations, delivering a list of promising organisms for the individual fermentation steps and giving an overview of the existing broad spectrum of products based on acetate and ethanol. Understanding of these pioneering approaches allows collecting ideas for new products and may open avenues toward making full use of the technological potential of these concepts for establishment of a sustainable biotechnology.

## Introduction

The worlds modern societies are built based on fossil energy sources, which are used to supply a myriad of products, to fuel global transportation systems and industries and to heat our homes. In consequence, land masses as well as oceans are increasingly polluted with industrial wastes and the atmosphere accumulates greenhouse gases in a dramatic velocity, which is tightly connected to climate change. Additionally, farmland soils are degenerating due to changing climates and excessive fertilizer usage, stoking a fuel vs. food debate. Without doubt, our life-style and our production processes must change as soon as possible toward green and sustainable solutions. One promising direction has been the use of C1 gases like CO, H_2_ plus CO_2_ or mixtures thereof (synthesis gas or syngas) as microbial feedstocks (Dürre and Eikmanns, 2015; Takors et al., 2018; Müller, 2019), which are often available as waste streams, e.g., as exhaust gases from gasification of biomass and solid waste streams (Stasiek et al., 2021), as industrial off-gases (Molitor et al., 2016), or as byproduct of combustion, thus circumventing food debates and going easy on dwindling resources (Dürre and Eikmanns, 2015).

**Figure 1:**
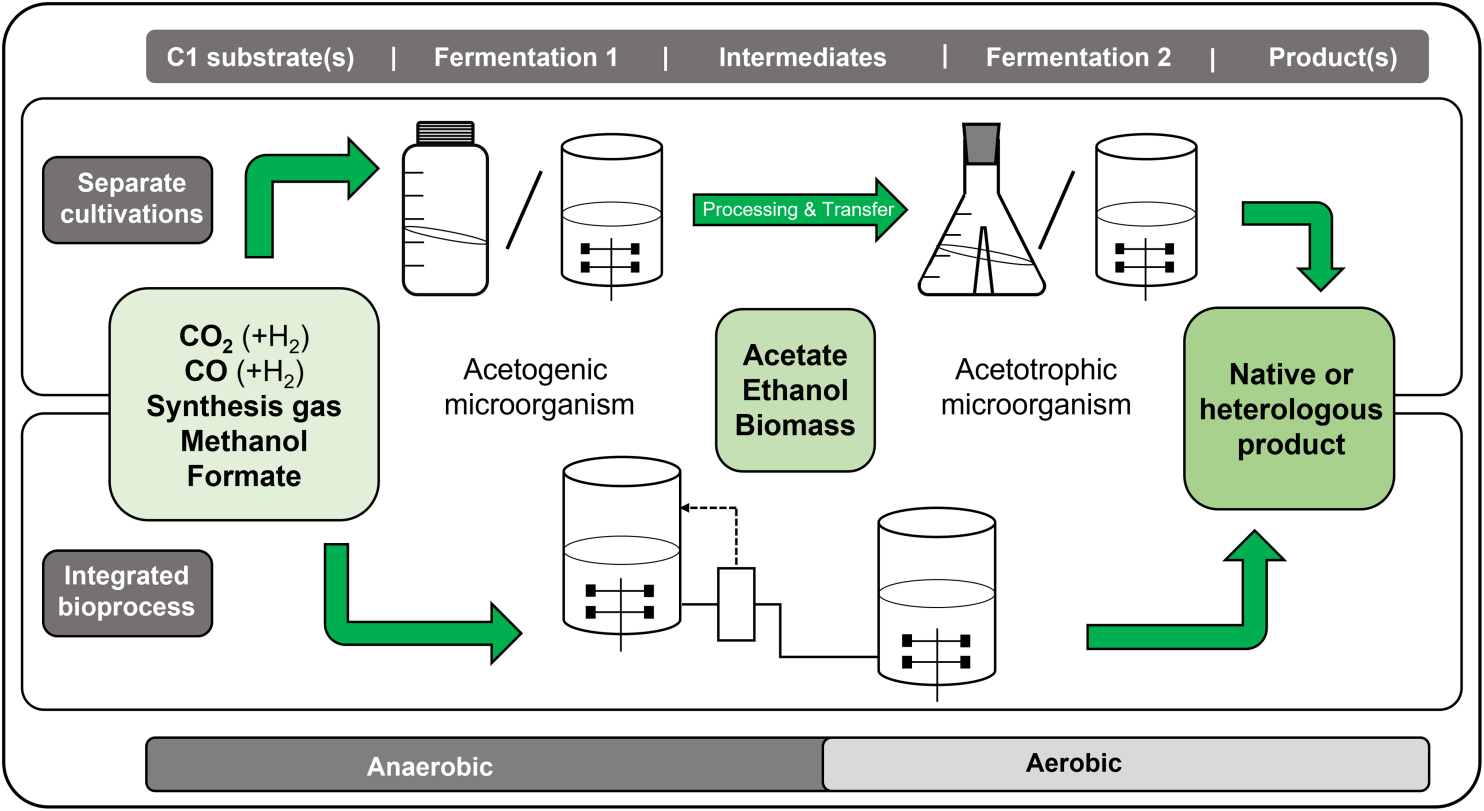
Schematic overview of the concept of sequential C1 fermentations for the conversion of C1 substrates, such as gaseous CO_2_, CO, mixtures like synthesis gas (syngas), or liquids like methanol or formate, toward higher products *via* a two-step fermentation process. In the first fermentation process, the respective C1 substrate is converted anaerobically by an acetogenic microorganism into acetate and/or ethanol. This/these intermediate product/s is/are further converted in a second, aerobic fermentation by an acetotrophic/ethanotrophic microorganism into the desired end-product, which can be a native or a heterologous product of interest. The synthesis of higher-value products from C1 substrates with sequential C1 fermentations can either be achieved with a process that separates both steps (upper part) or by combining both steps in an integrated process (lower part).

Acetogens as a group of anaerobic microorganisms can grow with the gaseous and liquid C1 compounds as carbon and energy source(s) forming biomass *via* the Wood-Ljungdahl pathway (WLP) and excreting acetate and/or ethanol as major metabolic end product(s) (Bengelsdorf et al., 2018; Müller, 2019). These microorganisms are strictly energy-limited and often require extensive metabolic engineering, but the absence of sophisticated genetic tools greatly restricts their engineering potential and product portfolio (Fast and Papoutsakis, 2012; Humphreys and Minton, 2018; Zeng, 2019). Nevertheless, their highly efficient acetate and/or ethanol production capability enables exploitation of the C1 compounds, since several established aerobic industrial hosts can grow with acetate and/or ethanol to produce native or heterologous products (Lim et al., 2018; Kiefer et al., 2020; Kutscha and Pflügl, 2020). Combining these “feeding” and “production” steps in sequential or integrated processes allows production of higher-value added and more energy-intense products descending from cheap and readily available C1 compounds. Such recent pioneering developments are exemplified in this article and summarized as “sequential C1 fermentations.” Common to them is that in a first anaerobic fermentation, acetogenic microorganisms convert C1 compounds to acetate and/or ethanol (Figure 1). In an intermediate step the medium containing this/these carbon source(s) is either adapted to the second microorganism or directly transferred into the second vessel. The aqueous intermediates are then fed to an aerobic microbe, which either produces a biotechnologically relevant homologous or heterologous product. Functional pairing of microbes rather than engineering organisms as C1 converting and production microbes appears to be advantageous since anaerobically growing acetogens usually (i) suffer from strict energy limitations and thus their achievable product spectrum is limited, (ii) are more difficult to engineer than industrial model organisms like, e.g., *Escherichia coli*, and (iii) grow much slower than most aerobes, which especially holds true for highly engineered hosts. However, the use of methanol, formate, acetate, and ethanol as substrates also has disadvantages, which will be discussed below. As outlined in recent reviews by Lim et al. (2018) and by Kiefer et al. (2020), in particular acetate has great potential to become a microbial platform substrate from which a myriad of products can be produced *via* (metabolically engineered) microbes (Kutscha and Pflügl, 2020), stepping in for sugar-based substrates.

## Sequential C1 Fermentations

Relatively few examples of sequential C1 fermentations with the gaseous C1 compounds CO_2_ plus H_2_, CO or syngas as original substrates in the first acetogenic fermentation and formation of “higher-energy products” have been described so far. In the following, we shortly describe these approaches, which mostly represent proof of principle studies. The sequential C1 fermentations presented in this review are all decoupled processes, C1 to acetate and then feeding of acetate-containing culture broth to the second fermentation. They slightly differ in the sense that they are separated either in time and space (Figure 1 upper part) or only in space (Figure 1 lower part). In both cases, the processes run in separate fermentation vessels, but in the latter case they run parallel in time, in which the fermentation broth from the first fermentation is continuously added to the second fermentation (designated as “integrated bioprocess”).

One of the first examples for sequential C1 fermentations has been described by Hu et al. (2016). These authors established and optimized an integrated bioprocess in a two-stage bioreactor system for continuous and efficient conversion of CO_2_/CO and H_2_/CO_2_ mixtures to microbial oil (triacylglycerides), that could be used as liquid biofuel. Accordingly, the system has been annotated as a novel biological gas-to-liquids (Bio-GTL) process by combining lipid synthesis in an engineered yeast with acetogenesis (Pfleger, 2016). In a first stage, the anaerobic thermophilic acetogen *Moorella thermoacetica* was employed for conversion of the gaseous substrates to acetic acid in a bubble column bioreactor. A hollow fiber membrane filter deployed in the anaerobic bioreactor allowed continuous removal of acetate-containing culture broth with simultaneous cell retention and recycling into the bubble column. The acetic acid broth from the first fermenter was continuously fed into a second bioreactor, containing an oleaginous yeast, *Yarrowia lipolytica*, which converted the acetic acid under aerobic conditions into lipids. As in the first stage of the integrated process the authors used hollow fiber membranes also in the second stage as well as recirculation pumps for cell recycling, to obtain high cell densities and high lipid concentration in the fermenter. The final titer of C16-C18 triacylglycerides was 18 g L^−1^, the productivity 0.19 g L^−1^ h^−1^, the lipid content of the *Yarrowia* cells was 36% of the dry weight. Although the whole process with these numbers is already efficient, the authors point out that the efficiency of their integrated system is lower than that of the individual bioreactor units and lower than theoretically expected. The overall energetic efficiency (from H_2_ to lipid and yeast) of the integrated system was 34.4% compared with the maximum theoretical value of 60.5%. Accordingly, the authors state that the process requires further optimization. However, the two-stage process presented by Hu et al. (2016) clearly showed net CO_2_ fixation and conversion of gaseous feedstocks to lipids.

A second example for sequential C1 fermentation was provided by Oswald et al. (2016), who sequentially coupled anaerobic syngas fermentation by the acetogen *Clostridium ljungdahlii* with malic acid production by the fungus *Aspergillus oryzae*, the latter using the acetate formed during energy metabolism of the acetogen. To meet the requirements for malic acid production by the fungus, an ammonia-reduced medium was used for both the syngas and the aerobic malic acid fermentation, the Bio-GTL process was conducted sequentially with a switch of fumigation conditions from anaerobic syngas to air. The authors found that acetate production by *C. ljungdahlii* started only when the medium was depleted of fructose, the carbon monoxide partial pressure was low and the organism started to produce H_2_. Moreover, they observed that malic acid production was less efficient when the acetogenic biomass was removed before starting the second fermentation step and concluded that the biomass may be used as source of nutrients and vitamins. The overall yield (Y_P/S_) for the conversion of CO and H_2_ into malic acid was between 0.12 and 0.22 g g^−1^, which is in the same range or even higher than that of anaerobic butanol production from sugar (Schiel-Bengelsdorf et al., 2013).

Starting with an optimized and waste-reduced syngas fermentation, Al Rowaihi et al. (2018) reported on a two-stage Bio-GTL process to convert CO_2_ into the bioplastic polyhydroxybutyrate (PHB). The authors used the pH-adjusted acetate-containing broth from an anaerobic *Acetobacterium woodii*-culture in a second fermentation for the synthesis of PHB under aerobic conditions, using *Ralstonia eutropha* H16 (nowadays designated as *Cupriavidus necator* H16). For the first fermentation (for up to 96 h), they used a sealed stirred-tank reactor at 2.0 and 5.5 bar pressure with pressure regulation, that prevented loss of gas and introduced fresh gas when the pressure in the reactor decreased as a result of gas consumption. In the second fermentation, 0.5 g PHB L^−1^ were produced from 3 g of acetate L^−1^, with a productivity of 0.1 g PHB L^−1^ h^−1^ and a PHB content of 33.3% within the cells. In an earlier study on polyhydroxyalkanoate (PHA) production from syngas as original substrate (Lagoa-Costa et al., 2017), *Clostridium autoethanogenum* was used to produce acetate and ethanol in the first step, followed by a second fermentation step using an enriched mixed culture, which converted the acetate to PHA. It should be noted here that *R. eutropha* has previously been shown to produce up to 61.3 g PHB L^−1^ with a productivity of 1.55 g L^−1^ h^−1^ within 40 h, when cultivated with CO_2_, H_2_ and O_2_, i.e., in an aerobic single-step gas fermentation (Ishizaki et al., 2001). The presence of H_2_ and O_2_, however, required an explosion-proof fermentation plant with security devices and countermeasures.

A sequential C1 fermentation in the format of a fed-batch process was set up by Lehtinen et al. (2018). With the aim of alkane production for use as drop-in fuel, the authors used acetate-broth from *A. woodii* cultures as batch- and feed-medium for recombinant *Acinetobacter baylyi* ADP1 strains. Relatively little attention and efforts were paid on the first step, the anaerobic production of acetate. The *Acinetobacter* strains used in the second fermentation were metabolically engineered to synthesize fatty aldehydes and converting these to alkanes by introduction and expression control of respective heterologous genes for aldehyde- and alkane-forming enzymes and deletion of native genes encoding alkane degradation enzymes. To avoid toxic effects of acetic acid and carbon limitation, a fed-batch process was chosen for alkane production. However, alkane productivity and final titers of the system remained relatively low, but the study represents a proof of concept and demonstrated that long-chain alkanes (e.g., heptadecane) can be produced from CO_2_ and H_2_ by sequential fermentation.

Yang et al. (2020) developed and optimized an *E. coli* isopropanol-production strain and cultivated it on acetate-containing broth from two different acetogens, *C. ljungdahlii* and *M. thermoacetica*, cultivated in the first fermentation in complex medium with syngas as main substrate. The *E. coli* strain employed in the second fermentation harboured heterologous genes for isopropanol biosynthetic enzymes from *Clostridium acetobutylicum* and *Clostridium beijerinckii* and it was genetically engineered to enhance acetate activation and NADPH availability by increasing the promoter activity for acetate kinase and phosphotransacetylase genes and introducing the NAD kinase and transhydrogenase genes. The best strain produced relatively low titers of isopropanol, however, the obtained yields (0.56 mol mol^−1^ acetate) were higher than the one theoretically expected (0.5 mol mol^−1^). This was explained by additional nutrients deriving from the complex medium of the acetogenic fermentation.

The last example for sequential C1 fermentations discussed here involved a syngas fermentation of *M. thermoacetica* and subsequent 3-hydroxypropionic acid (3-HP) production from syngas-derived acetate with simultaneous CO_2_-fixation using a recombinant *E. coli* strain (Lai et al., 2021). The strain carried codon-optimized genes encoding the enzymes for 3-HP synthesis from acetyl-CoA and was engineered for higher glyoxylate cycle activity, inhibited fatty acid synthesis and enhanced acetate activation. The 3-HP titers (11 g L^−1^) and yields (0.55 g g^−1^ acetate) obtained with syngas-derived acetic acid were similar to those obtained with chemically synthesized acetic acid. This result shows that the system applied had high efficiency for production of 3-HP from syngas-derived acetic acid with concomitant CO_2_ fixation in the second stage.

Table 1 gives a comprehensive overview on the most relevant data obtained from the given examples of sequential C1 fermentations.

**Table 1:** The first part gives an overview of the so far published approaches for sequential C1 fermentations, the second part lists acetogenic bacteria able to generate acetate and/or ethanol from C1 compounds.

Organisms	Process strategy	Substrate(s) and/or product(s)	Titer (g L^−1^)	Productivity (g L^−1^ h^−1^)	Product yield (Y_P/S_; g g^−1^)	Sources
**Sequential C1 fermentations**						
*Moorella thermoacetica* and *Yarrowia lipolytica*	Continuous mode	Syngas → acetate	30	0.57	-	Hu et al., 2016
	Fed batch mode	Acetate → C16–C18 triacylglycerides	46	0.27	0.16	
	Integrated continuous process consisting of BCR and STR	Syngas → acetate → C16–C18 triacylglycerides	18	0.19	0.09	
*Clostridium ljungdahlii* and *Aspergillus oryzae*	STR in batch mode	Syngas → acetate + ethanol	15.3 (Acetate) 0.6 (EtOH)	-	0.68	Oswald et al., 2016
	Shake flask	Acetate → malic acid	4.11		0.37	
	Separate processes, continuous mode/batch mode	Syngas → acetate → malic acid	1.83	-	0.22	
*Acetobacterium woodii*, and*Ralstonia eutropha* H16	High-pressure STR	CO_2_ + H_2_ → Acetate	4.5	0.05	-	Al Rowaihi et al., 2018
	Shake flask	Acetate → polyhydroxybutyrate		-	0.17	
	First high-pressure STR, then shake flask	CO_2_ + H_2_ → acetate → polyhydroxybutyrate	0.5	-	-	
*Acetobacterium woodii* and *Acinetobacter baylyi*	Fed batch mode	Acetate → alkane	0.54 × 10^−3^	0.021 × 10^−3^	-	Lehtinen et al., 2018
	Separate processes, continuous mode and fed batch mode	CO_2_ + H_2_ → Acetate → alkane	0.074 × 10^−3^	0.0082 × 10^−3^	-	
*Clostridium ljungdahlii*/*Moorella thermoacetica* and *Escherichia coli*	Separate processes, Fermentation in continuous mode and shake flasks	Syngas → acetate → isopropanol	1.47	-	0.56^*^	Yang et al., 2020
*Moorella thermoacetica* and *Escherichia coli*	Shake flasks	Acetate → 3-hydroxypropionate	15.8	-	0.71	Lai et al., 2021
	Separate processes, BCR and shake flask cultivations	CO_2_ + H_2_/syngas → acetate → 3-hydroxypropionic acid	11.2	-	0.55^**^	
**Microorganisms forming acetate and ethanol from C1 substrates**						
*Acetobacterium woodii*	STR in batch mode with continuous gassing	CO_2_ + H_2_ → Acetate	50.5	1.2	-	Straub et al., 2014
	SMBR	CO_2_ + H_2_ → Acetate	17.6	6.2	-	Kantzow et al., 2015
	STR in batch mode, continuous gassing	CO_2_ + H_2_ → Acetate	59	0.8	-	
	Anaerobe flasks	Methanol → acetate	-	-	-	Kremp et al., 2018
	Anaerobe flasks	Formate → acetate	2.8	-	-	Moon et al., 2021
			3.17	-	0.34	Neuendorf et al., 2021
	STR in continuous mode	Syngas → acetate	35.4	1.0	-	Novak et al., 2021
	Anaerobic flasks	CO + formate → acetate + ethanol	3.2 (Acetate) 0.2 (EtOH)	-	-	Bertsch and Müller, 2015
*Clostridium carboxidivorans* P7	Horizontal rotating packed bed reactor	Syngas → acetate + ethanol	6 (Acetate) 7 (EtOH)	0.2 (Acetate) 0.3 (EtOH)	-	Shen et al., 2017
	STR in batch mode	Syngas → acetate + ethanol	1.0 (Acetate) 2.5 (EtOH) and 0.6 (Acetate) 3.2 (EtOH)	-	-	Rückel et al., 2021
*Clostridium coskatii*	Anaerobe flasks	Syngas → acetate + ethanol	3.4 (Acetate) 0.1 (EtOH)	-	-	Flüchter et al., 2019
*Clostridium ljungdahlii*	STR in batch mode	CO_2_ + H_2_ → Acetate + ethanol	9.0 (Acetate) 0.1 (EtOH)	-	1.2 (Acetate) 0.02 (EtOH)	Hermann et al., 2020
	STR in batch mode	CO + H_2_ → Acetate + ethanol	0.7 (Acetate) 5.2 (EtOH)	-	0.04 (Acetate) 0.3 (EtOH)	
	STR in batch mode	Syngas → acetate + ethanol	0.38 (Acetate) 5.91 (EtOH)	-	0.01 (Acetate) 0.4 (EtOH)	
	Anaerobe flasks	Syngas → acetate + ethanol	~1.6 (Acetate) ~0.8 (EtOH)	-	-	Köpke et al., 2010
*Clostridium aceticum*	STR in batch mode	CO → acetate + ethanol	11 (Acetate) 0.35 (EtOH)	0.4 (Acetate)	-	Mayer et al., 2018
	STR in chemostat mode	CO → Acetate	2.7	0.32	-	
	SMBR	CO → Acetate	7.2	0.85	-	
	STR in batch mode, continuous gassing	CO → Acetate + ethanol	18 (Acetate) 4.4 (EtOH)	0.26 (Acetate)	-	Arslan et al., 2021
*Clostridium autoethanogenum*	Anaerobe flasks	CO → Acetate + ethanol	0.5 (Acetate) 0.4 (EtOH)	-	-	Abrini et al., 1994
*Thermoanaero-bacter kivui*	Anaerobe flasks	Syngas → acetate	4.7	-	-	Weghoff and Müller, 2016
*Eubacterium limosum*	Anaerobe flasks	CO_2_ + H_2_ → Acetate	5.0	-	-	Flaiz et al., 2021
	Anaerobe flasks	Syngas → acetate	3.9	-	-	
	Anaerobe flasks	Methanol → acetate	2.5	-	-	
*Moorella thermoacetica*	BCR with continuous gassing	CO_2_ + CO → Acetate	30	0.55	-	Hu et al., 2013
*Butyribacterium methylotrophicum*	Anaerobe flasks	CO + Formate → acetate	~0.6	-	-	Kerby and Zeikus, 1987
*Sporomusa ovata*	MES Reactor	Methanol → acetate	~0.9	-	-	Tremblay et al., 2015
	No information	CO_2_ + H_2_ → Acetate + ethanol	2.4 (Acetate) 0.07 (EtOH)	-	-	Ammam et al., 2016

-, no data available.

* g[Isopropanol]/g[acetate];

** g[3-Hydroxypropionic acid]/g[acetate].

Abbreviations: BCR, bubble column reactor; MES, microbial electrosynthesis; PHA, polyhydroxyalkanoate; PHB, polyhydroxybutyrate; PHBV, poly(3-hydroxybutyrate-co-3-hydroxyvalerate); SMBR, submerged membrane bioreactor; and STR, stirred tank reactor.

## C1 Fermentation: Potential Substrates and Organisms

For the first step of sequential fermentations, different gaseous or liquid C1 compounds, preferentially cheap and derived from non-food sources, can be used as substrates for acetogens. Hitherto, mainly syngas or CO_2_ was used (see Table 1), however, CO, formate, methanol and also methane (CH_4_) may be also promising C1 substrates. Syngas, CO_2_ and CO can be directly derived from industrial waste gases (steel manufacture, oil refining, coal and natural/shale gas) or produced by gasification and pyrolysis of solid waste or lignocellulosic biomass (Molitor et al., 2016; Takors et al., 2018; De Ras et al., 2019; Friedlingstein et al., 2019; Porshnov, 2021; Stasiek et al., 2021). H_2_, required as electron donor for reduction of CO_2_, can be provided electro- or photochemically with sustainable green power (Dincer and Acar, 2015). Formate and CO can be supplied by electro- or photochemical reduction of CO_2_ (Jouny et al., 2018; Li et al., 2019; Xiang et al., 2019) and by reacting H_2_ with CO_2_ (Álvarez et al., 2017). Methanol standardly is provided by the Fischer-Tropsch process but could also be supplied by reacting captured atmospheric CO_2_ and sustainably produced H_2_ (Szima and Cormos, 2018) or by direct conversion of CH_4_ (Park et al., 2019). CH_4_ is the main component in natural and shale gases and represents more than 50% of biogas generated from digestion processes and landfill sites and abundantly available. As a pure gas as well as constituent of natural gas it is discussed as a promising next generation carbon feedstock for the chemical industry (Conrado and Gonzalez, 2014; Fei et al., 2014).

During the last years, CO_2_, CO and syngas have been exploited as feedstocks for acetogenic bacteria and significant improvement in generating acetate and/or ethanol have been achieved (Lim et al., 2018; Table 1). From CO_2_ and H_2_, *A. woodii* was shown to produce up to 59 g acetate L^−1^ with space–time-yields of 148 g L^−1^ d^−1^ (Kantzow et al., 2015), *C. ljungdahlii* produced 9 g acetate L^−1^, plus smaller quantities of ethanol (Hermann et al., 2020), and *E. limosum* 5.0 g acetate L^−1^ (Flaiz et al., 2021). Interestingly, when CO was the main substrate for *C. ljungdahlii*, the product spectrum changed to 5.2 g ethanol L^−1^ and small quantities of acetate, 2,3-butanediol and lactate.

Despite being toxic to almost all living organisms, several acetogens have been reported to tolerate and even grow with CO (Diender et al., 2015). Prominent CO-utilizing acetogens comprise *A. woodii*, reported to produce acetate and small quantities of ethanol from CO and formate (Bertsch and Müller, 2015), *C. aceticum*, showing maximal titers of 18 g acetate L^−1^ (Arslan et al., 2021), and *M. thermoacetica*, reported to produce 31 g acetate L^−1^ (Hu et al., 2013).

Based on syngas of varying compositions, *A. woodii* has been shown to produce up to 35 g acetate L^−1^ (Novak et al., 2021), *Thermoanaerobacter kivui* showed titers of 4.8 g L^−1^ (Weghoff and Müller, 2016), *E. limosum* provided 4 g L^−1^ with small quantities of butyrate (Flaiz et al., 2021), and *C. coskatii* produced 2 g L^−1^ with small quantities of ethanol (Flüchter et al., 2019). Additionally, LanzaTech NZ Ltd. is known to produce ethanol with *C. autoethanogenum* in large scale (Köpke et al., 2011; Liew et al., 2016). Aside from this, larger scale production from syngas has not been reported yet.

Anaerobic CH_4_ oxidation to acetate has recently been shown with a recombinant *Methanosarcina acetivorans* strain expressing heterologous methyl-coenzyme M reductase genes from an archaeal population (Soo et al., 2016) and also with a culture containing mainly an anaerobic methanotrophic archaeon (Cai et al., 2019). These studies indicate that methane may also be used as an original feedstock for sequential C1 fermentations and thus also for production of higher-value products.

Beyond C1 gases, also the liquid C1 compounds methanol and formate have recently gained attention as potential substrates for acetogenesis (Müller, 2019; Cotton et al., 2020). Both have several distinct advantages, such as their higher solubility and better transportability (Yishai et al., 2016; Álvarez et al., 2017; Cotton et al., 2020), and they share low prices and availability from fossil and renewable sources (Yishai et al., 2016; Cotton et al., 2020). Methanol concentrations promoting best growth depend on the chosen host, but generally seem to be in the range of below 2% (v v^−1^), whereas concentrations of 4%–6% (v v^−1^) methanol completely inhibit growth (Cotton et al., 2020). *A. woodii* and *E. limosum* have been shown to grow with methanol as sole carbon source and to produce acetate (Kremp et al., 2018) or acetate plus small quantities of butyrate (Flaiz et al., 2021), respectively. With respect to formate as substrate, there are only very few studies on acetate/ethanol production with acetogenic microorganisms. *A. woodii* and *Butyribacterium methylotrophicum* were reported to produce mainly acetate when grown with either formate or formate plus CO_2_ and/or CO (Kerby and Zeikus, 1987; Bertsch and Müller, 2015; Moon et al., 2021; Neuendorf et al., 2021). However, comprehensive analyses and bioreactor studies about acetogenesis from methanol and formate are missing so far.

The use of formate or methanol implies several considerable technological and physiological challenges. Both exhibit cellular toxicity above certain concentrations, methanol toxicity being due to high reactivity of metabolically formed formaldehyde that is known to inactivate proteins (Pluschkell and Flickinger, 2002). Formic acid is a small organic acid and similarly to acetic acid, it crosses the cell membrane and dissociates intracellularly, dissipating into formate plus protons, thereby acidifying the cytosol (Warnecke and Gill, 2005). On the process side, handling of toxic substrates demands sophisticated cultivations techniques including fine-tuned fed-batch strategies and avoiding locally high concentrations, e.g., in large scale bioreactors. On the other hand, contamination risks can be minimized by using potentially toxic substrates such as methanol or formic acid (Cotton et al., 2020).

Besides the liquid C1 substrates also the acetogenic products (acetate/ethanol) are well known to pose challenges, which is addressed in the following chapter.

## Product Formation From Acetate and/or Ethanol: Potential Organisms and Products

The second step within sequential C1 fermentations is an aerobic process that enables production of more energy-intense, higher-value products from acetate and/or ethanol. Potential host cells must bring three main features: (i) a solid resistance against acetate and/or ethanol, which have been shown to be challenging as microbial feedstocks, (ii) uptake and metabolization of the intermediate products preferentially with solid growth rates, and (iii) either the capability of naturally producing a desired product from acetate and/or ethanol or to be accessible to genetic engineering for transformation into a desired production strain. Genetic/metabolic engineering is often used to improve acetate utilization and tolerance, to bring in new metabolic enzymes or pathways, and to enhance the productivity and product titer. Accordingly, products can be divided into native metabolites of the respective host cell and heterologous products of recombinant, genetically modified cells.

Acetate uptake and metabolism in prokaryotic and eukaryotic organisms and also the use of acetate as microbial feedstock for production purposes has gained increasing attention. This and a variety of examples of biobased transformation of acetate into value-added chemicals have thoroughly been described and discussed in recent reviews by Lim et al. (2018); Novak and Pflügl (2018); Kiefer et al. (2020) and Kutscha and Pflügl (2020). The herein described organisms employed for production from acetate were mostly metabolically engineered strains of *E. coli*, *Pseudomonas putida* and *Cupriavidus necator* and oleaginous yeast strains of *Yarrowia*, *Candida* and *Cryptococcus*. Among the products obtained from microbial conversion of acetate listed in the reviews cited above are organic acids (malic, succinic and itaconic acid and 3-HP), long-chain alcohols (isobutanol, isopropanol), hydroxyalkanoates (PHB, PHA), lipids (long-chain triacylglycerols, rhamnolipids) and proteins. Very recently, also recombinant *C. glutamicum* strains have been reported to produce 3-HP to concentrations of up to 17.1 g L^−1^ (Chang et al., 2022) and itaconic acid with titers of 29 g L^−1^ from acetate as sole carbon source (Merkel et al., 2022), the latter using an integrated pH-coupled feeding control.

As can be seen from the information given in the reviews cited above, the acetate-derived product spectrum is quite broad and this underlines the potential of acetate as an alternative platform substrate in the future biotechnology. However, the productivity and product titers of many of the products do not reach those of the primarily used carbon source. Reasons for that could be non-optimized process strategies, longer lag-phases (due to adaptation), and lower tolerance against acetate (see below). A further reason may be inefficient utilization of acetate by some of the organisms (Kiefer et al., 2020). The development of efficient bioprocesses and further efforts in the engineering of adjusted strains can enhance the productivity and product titer and enable the use of acetate in industrial range as platform substrate.

In contrast to acetate, which has been proposed as a strong alternative to sugar-based feedstocks, ethanol so far played a minor role as microbial feedstock. There are only few reports, e.g., on production of PHB, 3-HP, mevalonic acid with recombinant *E. coli* strains (Cao et al., 2020; Sun et al., 2020), of itaconic acid and PHB with *Saccharomyces cerevisiae* (Kocharin and Nielsen, 2013; Xu and Li, 2021) and of docosahexaenoic acid with *Crypthecodinium cohnii* (de Swaaf et al., 2003). Very recently, Yu et al. (2022) constructed *C. glutamicum* strains for recombinant protein production and secretion with ethanol as substrate. It is surprising that ethanol is not used more frequently because the conversion of ethanol to acetate generates additional NADH, that can be used for energy generation by respiration and thus should be advantageous for production purposes (Sun et al., 2020). Moreover, ethanol is a neutral molecule, which in contrast to acetate, does not have any influence on intracellular pH when taken up by cells (Trček et al., 2015).

It should be noted here that both acetate and ethanol have their challenges when used as microbial feedstocks (Wilbanks and Trinh, 2017). The deteriorating effect of acetate has been investigated intensely, it has been shown to mediate stress on the cell, including perturbation of anion pools, dissipation of the membrane potential, and acidification of the cytosol (Russell, 1992; Trček et al., 2015; Pinhal et al., 2019). Inhibition of microbial growth above certain concentrations resulting in prolonged lag-phases and lower growth rates or even no growth was reported for *C. glutamicum* and *E. coli* with acetate (Wendisch et al., 2000; Pinhal et al., 2019; Kiefer et al., 2020) and also with ethanol (Arndt et al., 2008; Cao et al., 2017). Ethanol is well known as cytotoxin that increases the permeability of the membrane for polar and charged molecules, leading to leakage (Ingram, 1989). However, such drawbacks can be tackled such as done with *C. glutamicum via* pH-coupled feeding of bio-acetate into the reactor (Kiefer et al., 2021), or with *E. coli* by rewiring the cAMP receptor protein (Chong et al., 2013) or by consecutive growth selections to increase tolerance (Sandoval et al., 2011). Beyond that, *E. coli* has been engineered in various ways to improve acetate uptake and productivity with acetate as carbon source (reviewed in Kutscha and Pflügl, 2020).

## Conclusion and Prospects

The novel concepts and developments summarized in this review, based on the intelligent creation of “food” and “production” bacteria and organized in a module style, enable the combination of the best of both the anaerobic and the aerobic world and are of outstanding biotechnological relevance. The bottom line of these processes is to simply let acetogens do what they do best—to produce acetate/ethanol from C1 bodies—and largely spare these fastidious anaerobic bacteria from genetic engineering and thus direct recombinant manufacture of high-quality products. Production is then carried out by well-established, aerobic bioengineering hosts, which also keeps these strains in the focus of their bioengineering capabilities. Among them, the prokaryotic *E. coli* and *C. glutamicum* and the yeasts *Yarrowia* and *S. cerevisiae* are most promising candidates for the efficient use of acetate and ethanol as potential next-generation platform substrates in industrial biotechnology. Current challenges and at the same time a highly dynamic field of optimization possibilities obviously lie in the biotechnological aspects of process development. Fundamental design must be carefully considered in order to obtain efficient, coupled processes, e.g., the reactor type(s) and the way “feeding” and “production” are linked. It should be noted here that in all examples of sequential C1 fermentations described so far CO_2_ is released by respiration and therefore, less CO_2_ is bound than with a theoretical direct conversion of CO_2_ or synthesis gas into products. A challenge will be to recycle the CO_2_ released by respiration in the aerobic production phase and to make it available again for the acetogens in the first anaerobic fermentation. Moreover, the efficiency of the sequential C1 fermentations is in general lower than that of the (optimized) individual fermentations (see Table 1). Also interesting is the question of whether acetogenic biomass has to be removed before feeding to provide cell-free acetate/ethanol, which in turn has to meet the requirements of subsequent production. This is undoubtedly demanding and requires a holistic view of both the technical concept and possibly even more demanding, the physiology of the microorganisms used.

A further critical point of sequential C1 fermentations is the overall H_2_ (or electron) efficiency, i.e., the efficiency of H_2_ utilization for the production of a desired product. Of our examples for sequential C1 fermentation, only Hu et al. (2016) reported that the overall energetic efficiency of their integrated system (from H_2_ to lipid and yeast) was significantly lower than theoretically possible (see above) and Al Rowaihi et al. (2018) showed energy efficiencies of 5%–55% for only the first fermentation (H_2_ to acetate), depending on gas pressure and the medium used. Thus, the situation with regard to the energy requirement for sequential C1 fermentation and the biotechnological utilization of C1 substrates is unclear and remains to be examined.

The division of labor in sequential C1 fermentations enables the production of chemicals and fuels from C1 waste gases of industrial processes, without consuming human or animal feed or high-value farmland. By directly consuming these problematic gases that would otherwise be released into the atmosphere, a real contribution is made to the environment by reducing greenhouse gas emissions. In addition, this transformation into a truly sustainable next generation of biotechnology may further strengthen the already significantly increasing social acceptance of consumer products from biotechnological processes replacing their petrochemical predecessors. This will turn out as a considerable contribution of biotechnology toward a global economy liberated from the long term ecologically and socio-economically destructive addiction to fossil resources.

## Author Contributions

BE and AS conceptualized the manuscript. CS, SM, FR, BE, and AS wrote, revised, edited, and approved the manuscript. All authors contributed to the article and approved the submitted version.

## Funding

CS is financed by the Nachwuchsakademie of the Graduate and Professional Training Center Ulm (ProTrainU). We acknowledge the financial support by the Open Access Publication Fund of University of Ulm for the article processing charge.

## Conflict of Interest

The authors declare that the research was conducted in the absence of any commercial or financial relationships that could be construed as a potential conflict of interest.

## Publisher’s Note

All claims expressed in this article are solely those of the authors and do not necessarily represent those of their affiliated organizations, or those of the publisher, the editors and the reviewers. Any product that may be evaluated in this article, or claim that may be made by its manufacturer, is not guaranteed or endorsed by the publisher.

## Abbreviations

BCR: Bubble column reactor
Bio-GTL: Biological gas-to-liquids
3-HP: 3-Hydroxypropionic acid
MES: Microbial electrosynthesis
PHA: Polyhydroxyalkanoate
PHB: Polyhydroxybutyrate
PHBV: Poly(3-hydroxybutyrate-co-3-hydroxyvalerate)
SMBR: Submerged membrane bioreactor
STR: Stirred tank reactor
WLP: Wood–Ljungdahl pathway
